# Case report: Endovascular embolization of a cerebral pseudoaneurysm caused by SARS-CoV2 infection

**DOI:** 10.3389/fneur.2022.991610

**Published:** 2022-10-04

**Authors:** Juan Antonio García-Carmona, Enzo von Quednow, Francisco Hernández-Fernández, Juan David Molina-Nuevo, Jorge García-García, María Palao, Tomás Segura

**Affiliations:** ^1^Department of Neurology, Santa Lucia University Hospital, Cartagena, Spain; ^2^Group of Clinical and Experimental Pharmacology, Institute for Biomedical Research of Murcia (IMIB), Murcia, Spain; ^3^Department of Neurophysiology, General University Hospital, Albacete, Spain; ^4^Unit of Interventional Neuroradiology, General University Hospital, Albacete, Spain; ^5^Department of Neurology, General University Hospital, Albacete, Spain; ^6^Department of Radiology, General University Hospital, Albacete, Spain; ^7^Medical School, Institute for Research in Neurologic Disabilities (IDINE), University of Castilla-La-Mancha, Albacete, Spain

**Keywords:** COVID-19, pseudoaneurysm, subarachnoid hemorrhage, embolization (therapeutic), CLOCCs, Guillain-Barre syndrome

## Abstract

**Background:**

Severe COVID-19 has been shown to produce convulsions, encephalitis, Guillain-Barré syndrome, or cerebrovascular disease. However, only 4 case reports described subarachnoid or brain hemorrhage caused by ruptured cerebral aneurysms or pseudoaneurysms in patients with COVID-19. Cerebral pseudoaneurysms represent <1% of all intracranial aneurysms and have been related to radiation therapy, vasculitis, rupture of true saccular aneurysms, arteriovenous malformations, and infections by bacteria and viruses, such as Epstein-Bar and Herpes virus.

**Case presentation:**

A 28-year-old Caucasian woman, with no medical history of interest and completely vaccinated against SARS-CoV-2, was admitted to Neurology due to progressive tetraparesis with areflexia, a cough, and a fever of 38°C. SARS-CoV2 PCR was positive while lumbar puncture, blood tests, and electromyogram showed criteria for Guillain-Barré syndrome. Despite the treatment, the patient developed dyspnea and tetraplegia requiring invasive mechanical ventilation. There was motor neurological improvement but a decreased level of consciousness was observed on day 13. A brain CT scan demonstrated an acute haematoma and cerebral arteriography showed a 4-mm pseudoaneurysm located in a branch of the left middle cerebral artery. Given the high risk of rebleeding, endovascular treatment was decided upon. Therefore, complete embolization of the pseudoaneurysm was carried out by using the synthetic glue N-butyl-cyanocrylate. Two days later, the patient was clinically and neurologically recovered and was discharged. Lastly, a new angiography showed no evidence of the pseudoaneurysm 3-weeks later.

**Conclusions:**

We report, for the first time, a patient suffering a severe immune reaction caused by SARS-CoV2 infection and developing a cerebral pseudoaneurysm treated with endovascular embolization without complications.

## Introduction

The SARS-CoV-2 infection is well-known for causing common symptoms such as fever, cough, fatigue, pneumonia, and severe acute respiratory distress syndrome (SARS), leading to multi-organ failure and death. Furthermore, severe COVID-19 has been related to the impairment of the nervous system in hospitalized patients producing convulsions, encephalitis, and Guillain-Barré syndrome (1). Finally, haemostatic function represents a complex interaction between the coagulation and fibrinolytic systems, platelets, and the vascular wall (2). This haemostatic function is impaired by the SARS-CoV2 infection (3), causing cerebrovascular disease (1).

Cerebrovascular disease occurs in 1–2% of hospitalized patients suffering from COVID-19 and it has been extensively reported as ischemic stroke, while a few cases of brain hemorrhage have been reported (4). Three case reports published subarachnoid hemorrhage caused by ruptured cerebral aneurysms with COVID-19 (5) and another case was reported in an adolescent suffering an intracerebral haematoma due to cerebral distal pseudoaneurysm rupture (6).

Cerebral pseudoaneurysms represent <1% of all intracranial aneurysms (7) and their treatment and management is a challenge. Pseudoaneurysms are characterized by complete disruption of the artery wall, resulting in an extravascular hematoma contained by just a thin layer of connective tissue (8). Thus, these lesions have been shown to cause intracranial hemorrhage in up to 60% of patients and 31–54% mortality (7, 8). Several causes have been related to the formation of intracranial pseudoaneurysms including radiation therapy, vasculitis, rupture of true saccular aneurysms, arteriovenous malformations, and infections (8). Infectious pseudoaneurysms have a slight preference for younger people, most of them are located in the anterior circulation and often multiple (8). They are known to be caused by bacteria, mainly *streptococcus* and *staphylococcus*, fungi, tuberculosis, and by viruses, such as the Epstein-Bar and Herpes viruses (7, 8).

Here, we report a case of an adult Caucasian woman suffering from COVID-19 with progressive respiratory symptoms, who developed a Guillain-Barré syndrome and a brain hemorrhage caused by a pseudoaneurysm.

## Case description

**Figure 1:**
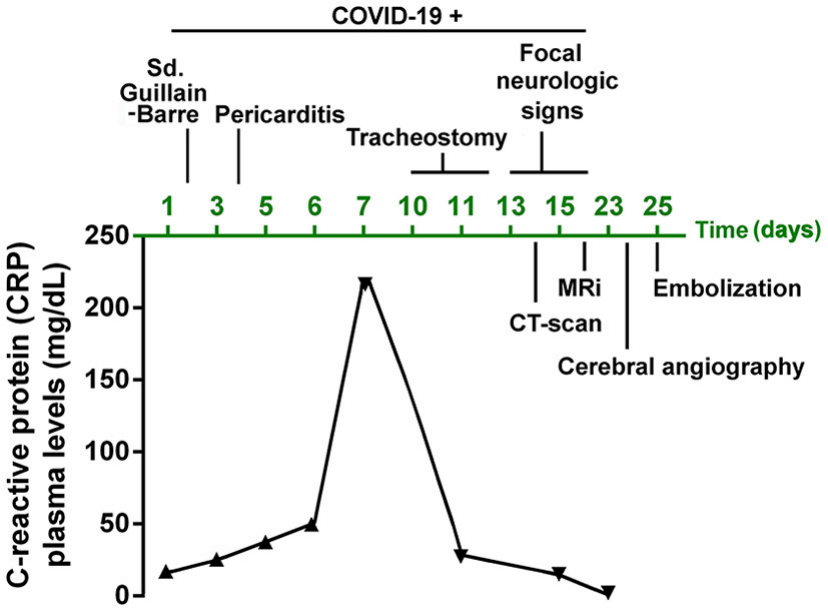
Timeline of the case report scheme summarizing the disease, complications and progression of the patient. Study follow-up of the C-reactive protein (CRP) plasma levels.

A 28-year-old Caucasian woman, with no medical history of interest and completely vaccinated against SARS-CoV-2 4 months ago, was admitted to Neurology due to a fever of 38°C and simultaneous progressive distal onset tetraparesis with areflexia. She also suffered mild respiratory symptoms, asthenia, and a cough from 1 week before (see Figure 1 for timeline scheme). No previous family history of autoimmune diseases, aneurysms, or stroke.

A chest X-ray showed bilateral pulmonary infiltrates and SARS-CoV2 PCR was positive. A lumbar puncture, blood tests, and an electromyogram (see Table 1 for details) were performed showing criteria for idiopathic acute demyelinating polyneuropathy or Guillain-Barré syndrome with positive IgG antiganglioside (GM1, aGM1, and GD1b) antibodies in relation to COVID-19 (day 2). Nonetheless, SARS-CoV2 subtype genotyping and PCR in cerebrospinal fluid (CSF) were not available. No other risk factors were identified underlying or related to Guillain-Barré syndrome (other viral or bacterial infections, recent surgery, hematological malignant diseases, or immune diseases such as lupus). Despite the patient being treated with immunoglobulins and corticosteroids she developed pericarditis on day 4 and progressed the polyneuropathy until she developed dyspnea and tetraplegia. No cranial nerve palsy was observed and neither was bowel or bladder dysfunction. Thus, she was transferred to the Intensive Care Unit (ICU), requiring invasive mechanical ventilation. As shown in Figure 1, neurological and respiratory worsening was in parallel with an increase in C-reactive protein (CRP) which peaked (>200 mg/dL) on day 7. As observed in a new chest X-ray, acute respiratory failure was probably secondary to a mucus plug causing airway obstruction and left lung atelectasis.

**Table 1:** Patient's demographic and clinical data.

Age and sex	28 y.o., woman
Ethnicity	Caucasian, Mediterranean European
Symptom prompting first neurological examination	Tetraparesis of distal onset (day 1)
Vital signs at admission	SBP/DBP 115/80 mmHg; HR 90 bpm; O2sat 95%
Sequence of findings	- COVID-19 pneumonia (day 1) - Guillain-Barre syndrome evidence (day 2) - Pericarditis (day 4) - Brain haematoma (day 14) - Pseudoaneurysm (day 15)
CSF biochemical, cytology and microbiologic test by PCR, culture and gram test	- Protein 26 g/dL, 3 cells, glucose 61 mg/dL - Cytology negative for malignant cells, isolated inflammatory cells - PCR NEGATIVE for *E. coli K1, Haemophilus influenzae*, Listeria monocytogenes, *Neisseria meningitidis, Streptococcus agalactiae and pneumoniae, Cryptococcus neoformans and gattii*, Citomegalovirus, Epstein-Barr Virus Enterovirus, Herpes simple virus 1, 2, 6, Parechovirusand Varicela-Zoster Virus. - Gram and long-term cultive for micobacteria and anaerobes negative
Antiganglioside antibodies	Antimonosialogangliosides GM1 = 1/8,907, GM2 and GM3 negative; antiaisialogangliosides GM1 = 1/18,189; antidisialogangliosides GD1b = 1/1,546, GD1a and GD3 negative; antitri- and tetrasialogangliosides GT1a, GT1b, GQ1b negative; antisulfatids negative.
EMG/ENG findings	Prolonged distal motor latencies and temporal dispersion of CMAP of bilateral peroneal and median nerves, complex A-waves of tibial nerves. Sensory nerves conduction studies were normal. No denervation signs were found. An acute inflammatory demyelinating polyradiculoneuropathy was diagnosed (AIDP).
Treatments	- Guillain-Barre syndrome: 0.4 g/kg/day intravenous immunoglobulin for 5-consecutive days - Pericarditis: colchicine 1 mg/day (for 3 months), Prednisone 0.5 mg/kg/day progressively decreased. - Pseudoaneurysm: endovascular embolization with N-butyl-cyanoacrylate
Recovery	- Guillain-Barre syndrome: 6 weeks - Pericarditis: 3 weeks - SARS-CoV2 pneumonia: 2 weeks

SBP, systolic blood pressure; DBP, diastolic blood pressure; HR, heart rate in beats per minute; O2sat, oxygen saturation; CMAP, compound muscle action potentials; ASA, acetylsalicylic acid.

**Figure 2:**
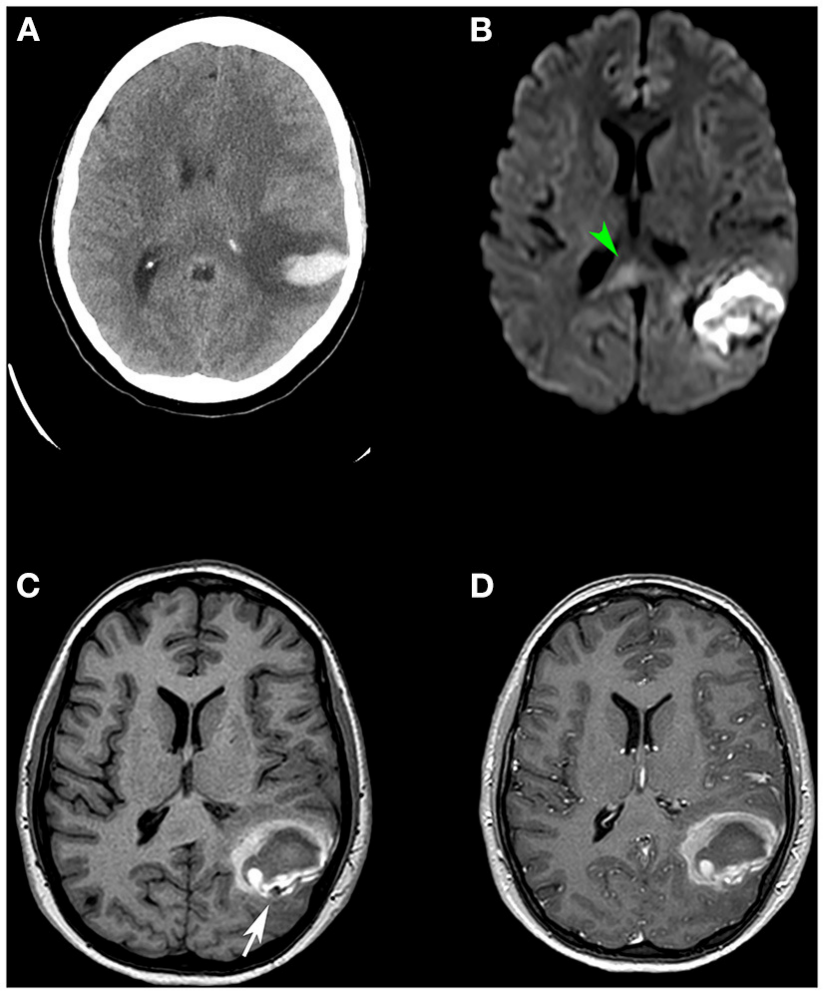
**(A)** Cranial CT scan showing left temporo-occipital intraparenchymal haematoma. **(B)** Diffusion-weighted MR image showing increased signal (diffusion restriction) and thickening of the splenium of the corpus callosum (green arrowhead), suggestive of a cytotoxic lesion of the corpus callosum (CLOCC). **(C,D)** 3D T1-weighted volumetric sequence without contrast **(C)** and with contrast **(D)** demonstrating the existence of a nodular image adjacent to the haematoma (white arrow). It presents as a signal void and after contrast administration, it enhances in an analogous way to vascular structures, compatible with a pseudoaneurysmal lesion.

On day 10, an elective tracheostomy was performed and sedation was suspended. Then, motor neurological improvement was observed on examination. Strikingly, a decreased level of consciousness and an absence of spontaneous language was observed on day 13. A brain CT scan (day 14) demonstrated an acute left parietal lobar haematoma (Figure 2A). It is worth noting that the patient was previously treated with a non-anticoagulant dose of enoxaparin 40 mg once/day (normal INR range in blood tests). A body CT and echography of the carotid, renal arteries, and heart were performed, finding no other aneurysms or pseudoaneurysms. Given these findings (and the arteriography results below), the absence of clinical features, and the lack of family history, other causes of aneurysms were reasonably discarded such as polycystic kidney disease, hereditary haemorrhagic telangiectasia, Ehlers-Danlos syndrome IV, Marfan syndrome, and neurofibromatosis I (7).

On day 18, an MRI confirmed the haematoma and showed a striking image of an irregular artery (Figures 2C,D). Interestingly, the MRI also revealed a non-specific focus of increased signal in the splenium of the *corpus callosum* (Figure 2B) on DWI sequences suggesting a cytotoxic lesion of the *corpus callosum* (CLOCC).

**Figure 3:**
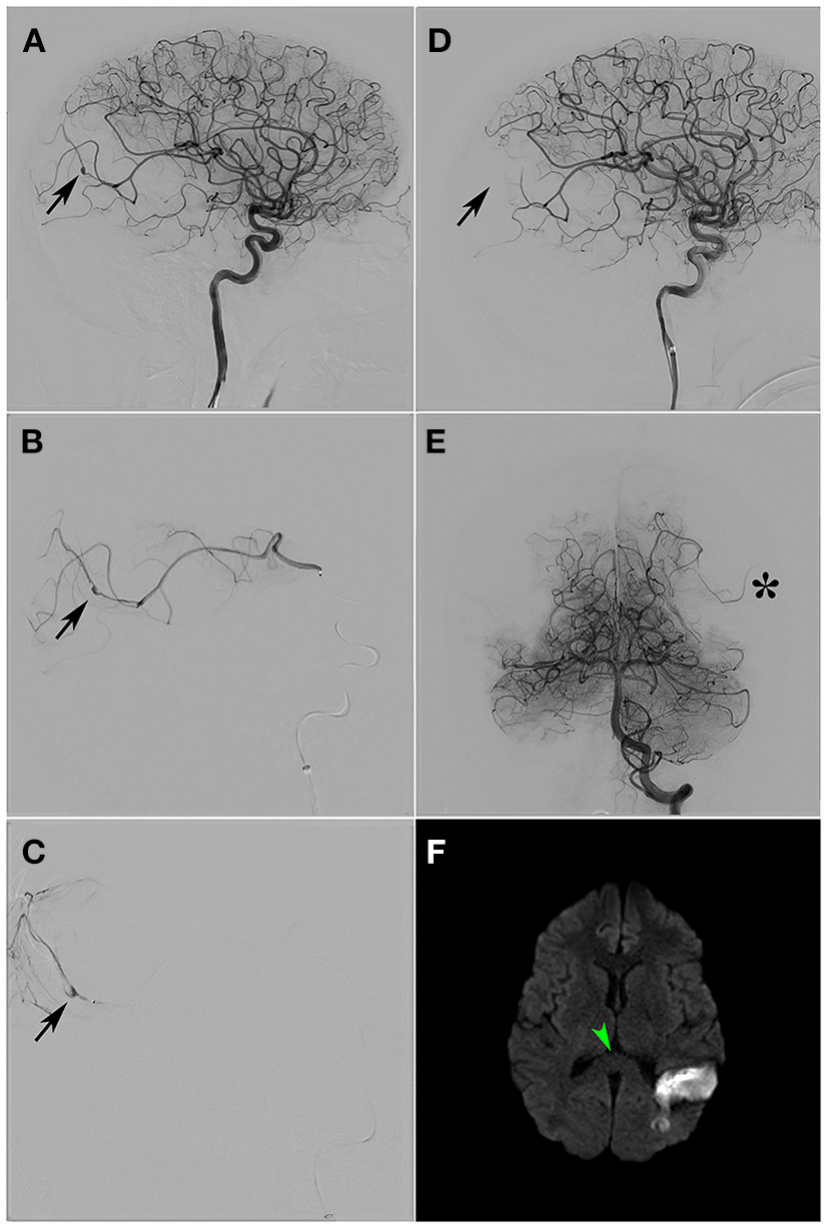
**(A)** Selective arteriography of RICA. The presence of a pseudoaneurysm is detected at the level of the distal temporo-occipital vasculature (black arrow). **(B)** Angular artery supraselective arteriography. The branch presenting the pseudoaneurysm shows an irregularity of the arterial wall suggesting arteritis (black arrow). **(C)** Glue cast after embolization (black arrow). **(D)** Selective arteriography of RICA after branch embolisation. Absence of pseudoaneurysm (black arrow). **(E)** Selective arteriography of LVA. Compensation of the left temporo-occipital territory through temporal branches of the left vertebral artery (black asterisk) is demonstrated. **(F)** Diffusion weighted MR image 1 month after treatment. Morphological and signal normalization of the splenium of the corpus callosum (green arrowhead). Absence of additional ischemic lesion after embolisation. RICA, Right internal carotid artery; LVA, Left vertebral artery.

After treatment and stabilization in the ICU, the patient presented clinical-neurological improvement (day 19). Then, diagnostic cerebral arteriography (day 24) showed a 4-mm distal pseudoaneurysm located in a branch of the temporo-occipital artery of the left middle cerebral artery (MCA), at the M4 level (Figure 3A). The parental artery of the pseudoaneurysm was a very thin caliber with irregular morphology, such as mycotic or vasculitic angiographic appearance (Figure 3B). Moreover, a hypoperfused left parietal lobe was observed, due to the mass effect of the haematoma, and a parieto-occipital artery from the left posterior cerebral artery (PCA) was compensating for the blood flow (Figure 3E). Given the high risk of rebleeding, endovascular treatment was decided upon. Therefore, complete embolization of the pseudoaneurysm (day 25) was carried out by sacrificing the parental artery by using the synthetic glue N-butyl-cyanocrylate (Glubran^®^, GME, Italy) through a 1.2F microcatheter (Magic^®^, Balt group, France) (Figures 3C,D). As expected, following the procedure, blood circulation was compensated by a parieto-occipital artery from the left PCA (Figure 3E). Two days later, the patient was clinically and neurologically recovered (excepting mild distal weakness in both hands) and was discharged from the hospital. A new MRI (Figure 3F) showed no ischemia, the CLOCC lesion had disappeared and the haematoma was reduced. Finally, a new angiography performed 3-weeks later showed no evidence of the pseudoaneurysm. At this point, motor function was completely recovered.

## Discussion

As previously reported, infectious pseudoaneurysms are often caused by bacteria or fungi and less frequently by viruses (8). COVID-19 has been reported as causing aneurysms and pseudoaneurysms in the heart and peripheral arteries (9–11). Nonetheless, only one case of cerebral pseudoaneurysm has been reported in an adolescent suffering from COVID-19 (6). This patient was diagnosed with a brain haematoma and a pseudoaneurysm in the left M2 segment. At this point, the patient had no respiratory symptoms caused by COVID-19 and the severe inflammatory or immune dysregulation took place 10 days after the treatment of the pseudoaneurysm by surgery.

In contrast, our patient suffered a severe immune reaction causing pericarditis and a severe Guillain-Barre syndrome with positive antiganglioside antibodies. Research on COVID-19 shows a prevalence of pericarditis of ~1.5% and it has been associated with increased odds of cardiac arrest, heart failure, and new-onset cardiovascular sequelae (12). Nonetheless, to the best of our knowledge, this is the first report of a patient suffering pericarditis and an intracranial pseudoaneurysm as COVID-19 complications. Moreover, in recent systematic reviews and meta-analyses, Guillain-Barre syndrome has been described in 1 per 10,000 cases of COVID-19 and antiganglioside antibodies have been reported in 0.8% of patients with Guillain-Barre and COVID-19 (13, 14). While few cases of Guillain-Barre and peripheral aneurysms have been reported, this is the first case reporting Guillain-Barre syndrome and an intracranial pseudoaneurysm related to COVID-19. No research has been conducted on antigangliosde antibodies and pseudoaneurysm formation. Furthermore, following the peak of the immune reaction, as suggested by the respiratory distress and the CRP plasma levels, our patient suffered a cerebral haematoma, and simultaneously, imaging tests demonstrated the presence of a CLOCC lesion and a pseudoaneurysm. CLOCCs have been described in a wide variety of conditions, including immune diseases, seizures, toxins, nutritional deficiencies, and infections such as the SARS-CoV2 as previously reported (15).

The SARS-CoV2 entry to the endothelial cell is facilitated by the binding of a spike protein to the angiotensin-converting enzyme-2 (ACE2) receptor leading to the inflammation and lesion of the artery wall through various mechanisms (16). Briefly, cell ACE2 internalization following SARS-CoV-2 entry impairs angiotensin 2 degradation and angiotensin 1–7 formation. Further, infected endothelial cells secrete cytokines and interferons which are recognized by their myeloid cell receptors mediating their migration and inflammatory activity. Moreover, spike protein exposure increases interleukins, MHC II, and costimulatory molecule (CD80 and CD86) expression by macrophages and dendritic cells, increasing the inflammatory and T cell-stimulatory activity (16). Finally, brain injury creates hypoxaemia, increasing proaneurysmal hypoxia-inducible factor (HIF)-1 levels. In this regard, previous studies have demonstrated that smooth muscle within the tunica media responds to localized hypoxia first by increasing HIF-1, resulting in the stabilization of the HIF-1α subunit, nuclear transcription, and binding to the hypoxia response element of VEGF and ets-1 transcription factor. The up-regulation of these genes induces the up-regulation of the matrix metalloproteinase (MMP)-2 and 9 and their increased protein levels. MMP-2 and 9 induce extensive artery matrix remodeling from tissue hypoxia (17). Therefore, we hypothesized that, through these mechanisms, SARS-CoV2 could cause aneurysms and pseudoaneurysms. Interestingly, evidence suggests that vascular dysfunction caused by COVID-19 manifests as deep venous thrombosis, embolism, and large arterial thrombosis. These manifestations are caused by hypoxaemia, viral sepsis, immobility, and vasculitis or vasculitis mimics (18, 19). Other cases of peripheral aneurysms and pseudoaneurysms have been reported (20, 21). These pseudoaneurysms secondary to COVID-19 are supposed to be secondary to inflammatory and vasculitis processes linked to viral multisystem inflammatory syndrome (18). Despite our radiological findings, the body CT scan and arteriography not being compatible with large/medium artery vasculitis means we cannot rule out that the formation of the pseudoaneurysm was caused by a multisystem inflammatory syndrome.

Several cases of endovascular treatment in pseudoaneurysms have been reported by using different approaches such as stents (22, 23), stent-assisted coils (24), embolic liquids/glues (25), and a case was even treated with a flow diverter device (26). In our case, we discarded the use of coils, stents, and flow diverter device because of the poor quality of the wall, thin artery caliber, and because the risk of haemorrhagic event was higher with the need for dual antiplatelet therapy. The reported case of pseudoaneurysm with simultaneous COVID-19 was treated with surgery (6). Nonetheless, we discarded that option to avoid significant periprocedural complications or poor outcomes as described (6).

In the present report, given the distal localization and the small caliber of the parental artery of the pseudoaneurysm, the evident collateral circulation from a parietal branch from the PCA, and the diminished parenchymal irrigation from the parental artery, we decided to sacrifice it by using embolic agents such as adhesive liquids and were successful.

## Conclusion

To the best of our knowledge, here we report for the first time, a patient suffering a severe immune reaction caused by SARS-CoV2 infection and developing a cerebral pseudoaneurysm treated with endovascular embolization without complications.

## Data availability statement

The raw data supporting the conclusions of this article will be made available by the authors, without undue reservation.

## Ethics statement

The studies involving human participants were reviewed and approved by Comité de Ética de la Investigación con Medicamentos de la Gerencia de Atención integrada de Albacete. The patients/participants provided their written informed consent to participate in this study.

## Author contributions

FH-F, JG-C, and EQ conceived the study and drafted the manuscript. FH-F, JG-C, EQ, JM-N, JG-G, MP, and TS acquired the data and critically reviewed the manuscript. All authors contributed to the article and approved the submitted version.

## Funding

This study was supported by the Asociación Médica para Investigación y la Docencia de Albacete (AMIDA).

## Conflict of interest

The authors declare that the research was conducted in the absence of any commercial or financial relationships that could be construed as a potential conflict of interest.

## Publisher's note

All claims expressed in this article are solely those of the authors and do not necessarily represent those of their affiliated organizations, or those of the publisher, the editors and the reviewers. Any product that may be evaluated in this article, or claim that may be made by its manufacturer, is not guaranteed or endorsed by the publisher.
